# Cryptococcosis and *Cryptococcus*

**DOI:** 10.1007/s11046-021-00577-7

**Published:** 2021-07-05

**Authors:** Elaine Cristina Francisco, Auke W. de Jong, Ferry Hagen

**Affiliations:** 1 Laboratório Especial de Micologia, Division of Infectious Diseases, Universidade Federal de São Paulo, São Paulo, Brazil; 2 Westerdijk Fungal Biodiversity Institute, Uppsalalaan 8, Utrecht, 3584CT The Netherlands; 3 Department of Medical Microbiology, University Medical Center Utrecht, Utrecht, The Netherlands

**Keywords:** *Cryptococcus neoformans*, *Cryptococcus gattii*, Diagnostics, Species complexes, Taxonomy, Treatment

Cryptococcosis is the collective heading of infections caused by members of the basidiomycetous yeast genus *Cryptococcus*, a notorious pathogen since the advent of the HIV/AIDS-pandemic. A cryptococcal infection usually manifests itself as a pneumonia and/or meningitis. The genus includes ten species, most of them belonging to the *C. gattii*/*C. neoformans* species complexes, and some non-pathogenic species (*C. amylolentus*, *C. depauparatus* and *C. luteus*) [1]. Previous rare causes of cryptococcosis are now accommodated in other genera (e.g. *Naganishia albida*, *Naganishia diffluens* and *Papilliotrema laurentii*) [1, 2].

*Cryptococcus neoformans *sensu stricto (previously *C. neoformans* variety *grubii*) is globally the major cause of systemic cryptococcosis among immunocompromised individuals. It is strongly associated with bird excreta, especially pigeon droppings [3]. *Cryptococcus deneoformans* (formerly *C. neoformans* variety *neoformans*) is less common but remain a major cause of cryptococcosis in Europe [3]. This applies to the interspecies hybrid *C. deneoformans* × *C. neoformans*, which occurs predominantly in Mediterranean Europe [3]. Relevant difference between *C. neoformans* and *C. deneoformans* is that the latter is associated with skin-infections and more often seen in elderly patients [3, 4].

**Fig. 1:**
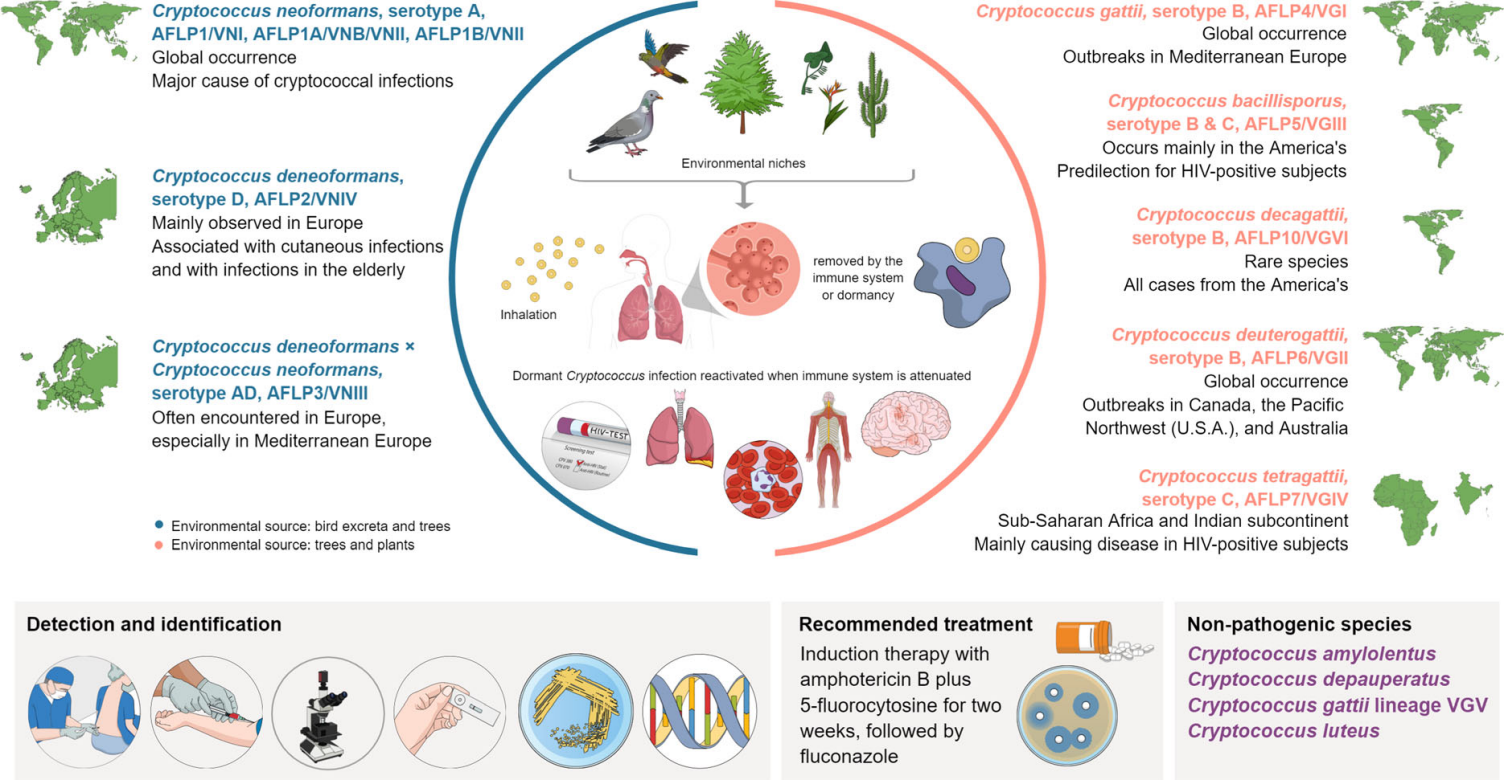
Characteristics of the pathogenic members in the *Cryptococcus gattii/Cryptococcus neoformans* species complexes. The upper part shows the 7 recognized species with serotype, AFLP genotype/molecular type, and geographic distribution. At the left side the *C. neoformans* species complex members, at the right side the *C. gattii* species complex members. Central to this are the environmental source and route of infection. The lower part depicts detection and identification methods, recommended treatment (based on [10]), and an overview of non-pathogenic species in the genus *Cryptococcus* (based on [1, 2, 6])

*Cryptococcus gattii *sensu lato (formerly *C. neoformans* variety *gattii*) became notorious due to the unprecedented outbreak on Vancouver Island (British Columbia, Canada). Until then, *C. gattii* was recognized as a pathogen exclusive to tropical and subtropical regions [5]. *C. gattii* is –like *C. neoformans*– a species complex, it comprises six lineages of which five are recognized as species while the sixth needs to be named [3, 6]. The ‘*C. gattii* VGII-outbreak lineage’ is now called *C. deuterogattii*. The environmental niches are trees and plants, and it is globally distributed like its sibling *C. gattii *sensu stricto. As both species occur in the environment of temperate climate zones it is obvious that they cannot longer be regarded as strict (sub)tropical pathogens. The remaining three pathogenic species have a predilection for the immunocompromised host. *C. bacillisporus* and *C. decagattii* infections are mainly reported from the America’s, with some inexplicable cases outside these continents [3, 5]. *Cryptococcus tetragattii* is, like *C. neoformans*, a frequent cause of cryptococcal meningitis among HIV-positive patients from sub-Saharan African and the Indian subcontinent (Fig. 1).

The cryptococcal polysaccharide capsule is an important virulence factor and an unmistakably aid in diagnostics. Cryptococcosis can be diagnosed by rapid and low-cost lateral flow assay, negative staining of CSF, culture, and molecular tools.

PCR-fingerprinting, AFLP genotyping, microsatellite typing, multi-locus sequencing typing and whole genome sequencing are widely used to investigate the molecular epidemiology [3, 6–9] (Fig. 1). The bipolar mating system, a set of genes with interacting *MAT****a*** and *MAT*α alleles, as well as mutation accumulation during clonal expansion, drives genetic diversity. From an epidemiological point-of-view (molecular) species-level identification is of importance and could contribute to adequate treatment.

Recommended treatment is a 2-weeks induction therapy with amphotericin B plus 5-fluorocytosine, followed by fluconazole as suppressive therapy [10]. *C. gattii *sensu lato infections need to be more aggressively treated than *C. neoformans *sensu lato*,* as the former produce cryptococcoma’s that are difficult to eradicate [10]. Antifungal susceptibility differences have been reported between species and it can be expected that in the future treatment is species-focussed rather than on the species complex [3].
